# Optimization of convolutional neural network and visual geometry group-16 using genetic algorithms for pneumonia detection

**DOI:** 10.3389/fmed.2024.1498403

**Published:** 2024-12-03

**Authors:** Mejda Chihaoui, Naziha Dhibi, Ahlem Ferchichi

**Affiliations:** ^1^Computer Science Department, Applied College, University of Ha'il, Hail, Saudi Arabia; ^2^REGIM: Research Groups on Intelligent Machines, University of Sfax, National School of Engineers (ENIS), Sfax, Tunisia

**Keywords:** pneumonia, deep learning, convolutional neural network, genetic algorithm, visual geometry group-16

## Abstract

Pneumonia is still a major global health issue, so effective diagnostic methods are needed. This research proposes a new methodology for improving convolutional neural networks (CNNs) and the Visual Geometry Group-16 (VGG16) model by incorporating genetic algorithms (GAs) to detect pneumonia. The work uses a dataset of 5,856 frontal chest radiography images critical in training and testing machine learning algorithms. The issue relates to challenges of medical image classification, the complexity of which can be significantly addressed by properly optimizing CNN. Moreover, our proposed methodology used GAs to determine the hyperparameters for CNNs and VGG16 and fine-tune the architecture to improve the existing performance measures. The evaluation of the optimized models showed some good performances with purely convolutional neural network archetyping, averaging 97% in terms of training accuracy and 94% based on the testing process. At the same time, it has a low error rate of 0.072. Although adding this layer affected the training and testing time, it created a new impression on the test accuracy and training accuracy of the VGG16 model, with 90.90% training accuracy, 90.90%, and a loss of 0.11. Future work will involve contributing more examples so that a richer database of radiographic images is attained, optimizing the GA parameters even more, and pursuing the use of ensemble applications so that the diagnosis capability is heightened. Apart from emphasizing the contribution of GAs in improving the CNN architecture, this study also seeks to contribute to the early detection of pneumonia to minimize the complications faced by patients, especially children.

## 1 Introduction

Over the last few decades, integrating machine learning and deep learning into medical imaging has initiated a revolution in healthcare (1, 2). As the diagnostics of various diseases largely rely on X-rays, CT scans, and MRIs, the amounts of data produced by these machines require further analysis with the help of complex tools and methods (2, 3). Routine diagnostic approaches requiring hand analysis are prone to limitations posed by human factors such as fatigue and variation of deftness. In this regard, the deep learning approach, especially CNNs, has been well-established to support the diagnostic process by automating and improving it. Such approaches help optimize healthcare practitioners' work and enhance diagnostic capabilities and velocity, potentially saving people's lives (4, 5).

CNNs are intended to learn feature hierarchies and extract spatial features from the input images as the network depends flexibly on the dimensionality of the input and is primarily employed for image-related tasks (6). Their architecture imitates the features of the human vision system to recognize multiple structures and patterns present in medical images. This capability has made CNNs especially helpful in identifying many medical conditions; pneumonia, for instance, is still prevalent and affecting the world today (7, 8). Diagnosing pneumonia through imaging is important since the disease can be well-managed if diagnosed early. Recent developments in CNN architecture, including the VGG model 16, mean that the performance of such systems is even higher because deeper networks can understand more complex representations of images (8). However, the problem of CNN optimization emerged as a rather costly affair, even though CNNs have capable features to deal with such a problem. However, the performance of a CNN depends on architectural design, parameters, and the quality of training datasets. Hence, anything that improves these parameters can be recommended for the best possible accuracy, as seen in the pneumonia detection tasks (9). The application of traditional optimization methods is effective. Still, the process frequently involves experimentation and domain knowledge, which can be highly time-consuming and may involve a massive deployment of resources. This is where genetic algorithms (GAs) are of great interest as they offer strategies to help achieve the above goal (10). A characteristic feature of GAs as adaptive search algorithms based on heuristics is their ability to effectively search the space of CNN architectures and hyperparameter combinations that have not been comprehensively studied before. Using selection, crossover, and mutation, GAs can find ideal or near-ideal solutions much faster than the traditional approach and at a minimum cost.

The use of GAs to enhance the performance of CNNs, including the VGG-16, concerning the prediction of pneumonia is a desirable innovation that could be pursued by Kör et al. (11). The use of gas in this area can subsequently enhance the model under test, thus causing a better distinction of pneumonia from radiographs or CT images. This is especially so considering the increasing number of studies showing that the applications of a machine learning model may outperform human specialists in precise diagnostic duties. Also, while optimizing, we can control overfitting, which is a common problem in deep learning, and thus ensure that the models are more generalized in new datasets (11, 12). This is important for the practical use of the developed method for detecting disease since differences in patient characteristics or imaging conditions may cause variations in the results. Moreover, the fact that CNNs may be tuned via GAs opens the discussion toward the increased popularity of individualized approaches to patient care and diagnostic or therapeutic management strategies. In using GAs, medical professionals can benefit from DL while simultaneously overcoming the problems arising from various diseases and patient datasets (12). Such customized improvement may improve the dependence of clinical operations on sophisticated programmed learning procedures and lead to increased acceptance among healthcare industry members. In addition to new technologies, the global aspects of pneumonia as the third cause of death overall and of childhood and elderly mortality, in particular, stress the need for developing better diagnostics (13). The World Health Organization says that pneumonia kills many people every year, and hence, there is a need for effective diagnostic methods. Incorporating refined deep learning models as work-horse systems in clinical workflow could dramatically change how care is delivered to patients with pneumonia to improve their health status (14).

**Figure 1:**
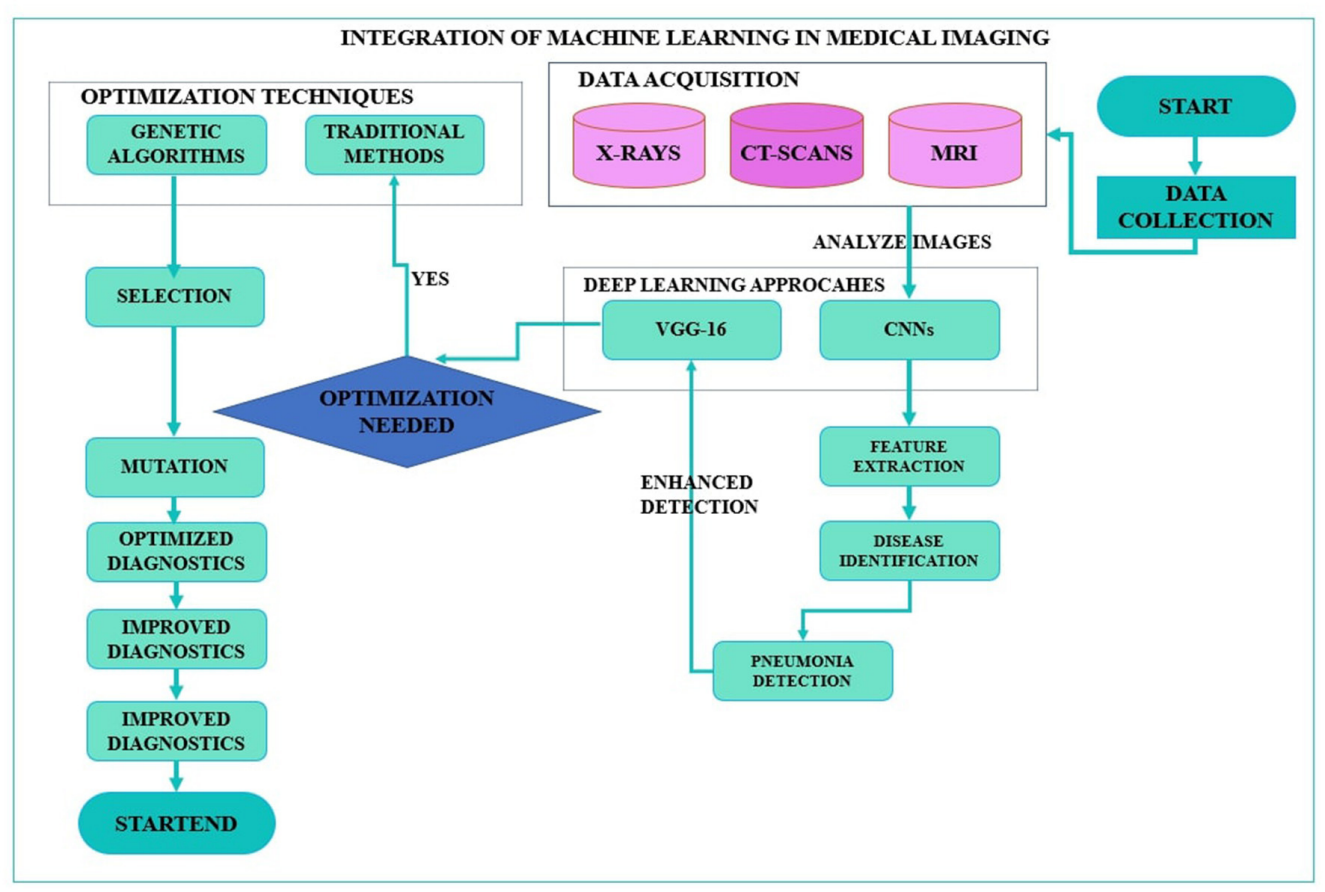
Framework of optimization of convolutional neural network and Visual Geometry Group-16 using genetic algorithms for pneumonia detection.

With increasing trends of quick and accurate diagnostic medical solutions, so is the importance of automated systems such as CNNs bolstered by inventive optimization methods such as GAs. Integrating these state-of-the-art approaches solves many current healthcare problems and forms the foundation of what is to come in medical imaging and diagnostics (15). The envisioned research study focuses on this integration process and investigates to what extent CNN architectures can be enhanced for pneumonia detection via the genetic algorithms application, thus advancing the discussions on using artificial intelligence in healthcare. Furthermore, the application of genetic algorithms for the optimization of CNN, specifically VGG-16, to detect pneumonia is a unique, innovative idea in this area of integration of artificial intelligence in healthcare. Figure 1 shows the proposed framework, whereby genetic algorithms are used to enhance both the CNN model and VGG-16 structure in the detection of pneumonia. This approach optimizes the identification of highly appropriate hyperparameters, improving classification and avoiding overtraining, especially in medical imaging applications.

The following are the contributions of this study:

- Enhanced model performance: The findings show that the proposed technique is robust in enhancing the performance of pneumonia detection by tuning CNN architecture and VGG-16 with genetic algorithms and improving doctors' diagnostic tools.
- Automated hyperparameter tuning: The study presents an effective method for hyperparameter optimization using genetic algorithms. This method saves time and training mandates necessary to set up complicated neural network architectures and thus enhances the model development procedure.
- Robust feature selection: This study focuses on the use of feature selection in the genetic algorithm form and identifies essential features that can boost the model's capacity to distinguish between pneumonia and healthy status, thus eradicating poor clinical decisions.
- Scalability and generalization: The current study's results show that the optimized models generalize well-across different datasets, a characteristic imperative for application in numerous clinical settings.
- Contribution to medical imaging research: This study contributes to the extant literature in the medical imaging domain by presenting a novel approach that combines genetic algorithms with a deep learning method. This approach points out potential directions for future research focused on automating and enhancing diagnostic procedures in healthcare.

The rest of the study is organized as follows: Section 2 provides a literature review on the topic. Section 3 outlines the procedure for detecting pneumonia. Section 4 describes the experimental results, and Section 5 provides an extensive discussion of the results. Last of all, Section 6 is the conclusion of the study, reflecting upon its limitations and opening the possibility for further research studies. Such structure allows for a comprehensive analysis of pneumonia identification methods and indicates the proposed approach's advantages.

## 2 Literature review

In medical image analysis, CNNs have attracted immense interest, especially in detecting pneumonia from chest X-ray images. CNN architectures have been at the forefront of enhancing diagnostic accuracy, and numerous investigations have considered diverse approaches to enhancing these frameworks. Among the techniques utilized, genetic algorithms (GAs) turn out to be a promising way to improve the performance of CNNs through the regulation of their hyperparameters and architectures. This literature review will also visualize all the different research papers, techniques, and datasets associated with pneumonia detection and the possibilities of GAs to enhance the CNNs, especially the VGG-16.

Mabrouk et al. (16) worked on an ensemble learning model-based model that incorporates DenseNet169, MobileNetV2, and Vision Transformer to utilize the ImageNet dataset. Such drug repurposing results are promising as they substantiated the high accuracy statement of 93.91% and F1-score of 93.88%. However, they agreed that their ensemble method could incorporate some biases and variance, which stated that hyperparameters must be meticulously tuned and pre-trained. This issue highlights a common challenge in CNN optimization: the problem of model complexity and the problem of model generalization. Likewise, Lamia and Fawaz (17) created a smart mobile application supported by the neural network, which was trained using a database involving more than 5,000 real images. Validation results in 97% accuracy, but the current accuracy in the test amounts to 86%. This means that there are some weaker points in the model, and the most significant of them can be clearly seen if the model is used in a mobile environment, where there are many limitations to computational power and model size. These difficulties highlight the need for other optimization strategies to enhance the proposed model's consistency across the platforms. Moreover, Sharma and Guleria (18) dealt with the inconsistency in X-ray interpretations by using VGG16 in combination with neural networks in two different datasets of Kaggle. Both of them achieve the accuracy rates of 92.5 and 95.4%, respectively. However, having noticed objective and subjective variability in the results of X-ray interpretation, they emphasized that it had high effectiveness of the method but could cause discrepancies in the diagnosis. This volatility indicates that although deep learning can lead to increased diagnostic efficiency, it cannot eliminate human evaluation and indicates that there is a need for the development of models that incorporate machine learning with expert feedback. The study Pranaya et al. (19) , where the authors worked on ~5,863 chest X-ray images from Kaggle, used CNN for feature extraction through convolutional layers with ReLU activation and max pooling. Their proposed approach achieved 91% accuracy as a result; however, they noted that in practice, hyperparameter tuning and expert assessment would be required for accurate diagnosis. We take this as an indicator of the need for further speed improvement and optimization in deep learning models to improve diagnostic ability. Another important study was produced by Reshan et al. (20) who performed the chest X-ray image analysis using datasets containing 57,573 and 60,515 images, over 117,000 images, utilizing the MobileNet-based deep learning model. For this, they found that their proposed approach had an accuracy of 94.23 and 93.75% for the respective datasets. However, they found that differences in the methods of acquiring X-ray scans could also affect the algorithm, suggesting that there is a need for dataset compliance with a myriad of imaging conditions. Moreover, Aljawarneh and Al-Quraan (21) used another dataset of 5,863 chest X-ray images, using VGG19 and ResNet 50 with improved CNNs. With this improved CNN model, the authors claimed an accuracy of 92.4% in correctly identifying the animals but cautioned that restricted choice of subjects could hinder model transferability. These are consistent limitations that point to absolutely crucial research directions in the future, primarily related to the fact that the dataset could and should be more diverse, and its expansion would bring the best results and would help to build a model that works effectively in various populations and with various types of images.

Furthermore, Miguel et al. (22) had a different approach whereby they developed a neural network that was trained with an evolutionary algorithm and was applied to histological images. Their model obtained up to a 0.71 AUC score, defining the moderate classification model efficacy. This study also points toward the future application of incorporating evolutionary algorithms into the training of a model, specifically the inclusion of additional performance metrics beyond basic accuracy rates. Similarly, Ismail et al. (23) enhanced a more specific approach of layer pruning with genetic algorithms to optimize CNN-based models in considering CT scans and ECG data connected with COVID-19. Their findings showed they achieved high hit ratios of 98.48% for MobileNet-V2 and 99.65% for VGG-16. The presented work of applying genetic algorithms in model pruning is a breakthrough toward the enhanced optimization of deep learning architecture for improving computational efficiency with minimum loss of accuracy. In addition to this, Sitaula and Aryal (24) made COVID-19 chest X-ray datasets accessible, thereby implementing the Bag of Deep Visual Words (BoDVW) technique. The work reported by these authors found accuracy scores in the range of 82.00–87.92% for different datasets. Nevertheless, issues such as restricted datasets and possible dataset overfitting were still prevalent, indicating the significance of creating new model variations and choosing datasets carefully. In addition, to counteract dataset biases, Balamurugan and Balamurugan (25) used the DARUNDNN model with the Dingo Optimization Algorithm on several real datasets, Montgomery as well as Shenzhen. They established that their results had very high specificity levels and near an accuracy of 99%. However, similar to previous experiments, the problem of biases in the datasets used for training and generalization problems also continued, which indicates the requirement for more precise and diverse datasets. Furthermore, Shuaib et al. (26) also zoomed into the ChestXray14 dataset and embarked on work to build a web application for pneumonia detection. They pointed out that relying strictly on frontal radiographs, as some of them have indicated, may lead to a reduction of diagnostic accuracy due to the need to obtain lateral views in some cases. Their model reached an 84% accuracy, and the authors pointed out that the utilized approach shows the limitation of the current methodological frameworks and stresses the necessity of strict imaging protocols. Last of all, Venu (27) used transfer learning on 5,856 Chest X-ray image datasets and got an outstanding accuracy of 98.46%. However, they failed to compare their results with the overfitting problem or specific dataset information. This void also emphasizes the need to conduct enduring evaluation frameworks for machine learning programs.

Chhabra et al. (28) used color masks and stacked autoencoders with the IIIT-D latent fingerprint dataset with a 98.45% segmentation accuracy in high-quality images but 14% less in low-quality images. Furthermore, Sharma et al. (29) applied a GAN-CNN model for deepfake detection, achieving 98.67% training accuracy but only 70.08% testing accuracy, showing variation in the curriculum. Moreover, Jeribi et al. (30) employed DLEF-SM with CNNs for forecasting of stock markets where the accuracy rate obtained was above 99%. In conclusion, numerous studies have established the applicability of CNNs and related approaches for pneumonia detection; however, multiple issues are still to be solved, such as the model's generalization, variation in the interpretation of the images, or the need for various datasets. Based on the analysis provided, genetic algorithms seem to offer a feasible method of solving the optimization problem associated with CNN architectural designs concerning hyperparameters and model reduction. There is a need to explore research that implements GAs more closely to CNNs in the future, along with improvements to the dataset variety to increase the dependability of the models when used in medical practice. To overcome these disadvantages, researchers can assist in developing better and stronger diagnoses for the fight against pneumonia and many other illnesses. Table 1 shows the list of past references, including datasets, methodology, limitations of the work, and results.

**Table 1:** Existing pneumonia detection approaches.

**References**	**Dataset**	**Methodology**	**Advantage**	**Limitations**	**Results**
Mabrouk et al. (16)	ImageNet	CNN Ensemble Learning (EL) using DenseNet169, MobileNetV2 and Vision Transformer	Combines multiple models for improved performance.	The suggested EL approach should have a lot of bias and variance. Specifying the pre-trained CNN techniques' hyperparameters while using TL and fine-tuning.	Accuracy = 93.91% F1-score = 93.88%
Lamia and Fawaz (17)	A dataset containing more than 5,000 real images	A mobile application that uses a neural network	User-friendly mobile application for diagnosis.	A developed model was trained with two categories (normal and Pneumonia), so any image was classified into one of these two groups.	Accuracy = 97% for validation and 86% for test. accuracy = 85% on a mobile platform
Sharma and Guleria (18)	Two CXR image datasets were taken from Kaggle	The VGG16 with Neural Networks	High accuracy in detecting pneumonia cases.	Subjective variability in X-ray interpretation affects detection accuracy.	D1 (accuracy = 92.5%, recall = 0.930, precision = 0.94, F1-Score = 0.93) D2(acc = 95.4%, recall = 0.95, precision = 0.954, F1-Score = 0.95)
Pranaya et al. (19)	Approximately 5,863 chest X-ray images from Kaggle	CNN to extract image features using convolution ReLU and max pooling.	Effective feature extraction for improved results.	Hyperparameter settings and Expert assessment are still required for reliable diagnosis	Accuracy of 91%
Reshan et al. (20)	Two publicly available datasets, including 112,120 and 5,856 chest X-ray images	DL model using MobilNet	High accuracy across diverse datasets	Variability in X-ray acquisition affects algorithm performance.	Accuracy = 94.23% for D1 and 93.75% on for D2
Aljawarneh and Al-Quraan (21)	Approximately 5,863 big chest XRIs from Kaggle	Enhanced CNN, VGG19, ResNet-50, and ResNet-50	Utilizes multiple architectures for flexibility.	Limited dataset diversity may affect model generalization.	ResNet-50: acc = 82.8%, enhanced CNN model: acc = 92.4%
Miguel et al. (22)	Histological images stained by hematoxylin-eosin.	Neural networks are trained using evolutionary algorithms.	Evolutionary methods enhance model training.	AUC values indicate moderate classification performance.	Achieved maximum AUC of 0.71.
Ismail et al. (23)	CT-scan images and ECG recordings of COVID-19.	Selective layer pruning with genetic algorithm for fine-tuning.	Achieves high accuracy with fine-tuning.	Computational complexity in optimizing pre-trained models.	Accuracy of 98.48% for MobileNet-V2 and 99.65% for VGG-16.
Sitaula and Aryal (24)	Publicly available COVID-19 CXR image datasets (D1, D2, D3, and D4)	Bag of Deep Visual Words (BoDVW)	Innovative approach to image classification.	Limited dataset diversity and potential overfitting issues persist.	Accuracy of 82.00% on D1, 87.86% on D2, 87.92% on D3, and 83.22% on D4)
Balamurugan and Balamurugan (25)	Montgomery, Shenzhen, and National Institutes of Health CXR.	Pre-processing, DARUNDNN model, Dingo Optimization Algorithm application	High accuracy with optimized algorithm.	Dataset bias and potential generalization issues remain unaddressed.	Shenzhen's accuracy is 98.92%, Montgomery's accuracy is 98.982%, and there is high specificity.
Shuaib et al. (26)	ChestX-ray14, publicly available	A web application-based DL utilized to detect pneumonia	Accessible web application for pneumonia detection.	Only frontal radiographs were used. However, up to 15% of accurate diagnoses required the lateral view. The model and the radiologists were not permitted to use patient history	Accuracy = 84%
Venu (27)	Chest X-ray dataset of 5,856 chest X-ray images	Transfer learning to reduce the neural network training	Reduces training time with effective results.	Dataset details and potential overfitting not addressed explicitly	Accuracy = 98.46%, precision = 98.38%, recall = 99.53%, F1-score = 98.96%
Chhabra et al. (28)	IIIT-D database of latent fingerprint images.	Hybrid approach using color masks and stacked autoencoders.	High segmentation accuracy through enhanced feature extraction.	Performance may decline on poor-quality fingerprint images.	Achieved 98.45% segmentation accuracy on good-quality images.
Sharma et al. (29)	Diverse deep fake images for training and testing.	GAN-CNN model using generative replay for detection.	Minimizes catastrophic forgetting for robust deep fake detection.	Performance varies with different types of deep fakes.	Achieved 98.67% training accuracy, 70.08% testing accuracy.
Jeribi et al. (30)	Historical stock market data for S&P500 and DAX.	DLEF-SM uses CNNs, optimization algorithms, and reinforcement learning.	High forecasting accuracy with advanced pre-processing and selection.	Complexity may hinder implementation for smaller datasets.	Achieved 99.562% accuracy for S&P500-S, 98.235% for S&P500-L.

## 3 Overview of the proposed approach

The idea of enhancing CNNs and VGG16 models through a new approach based on GAs for pneumonia detection involves the application of cutting-edge approaches to improve diagnostic efficiency. CNNs have made a huge improvement to the type of image analysis given their capability to map hierarchical representations of data and thus are effective in tasks such as object detection and classification. However, several inherent limitations can reduce their efficiency, such as noisy data sensitivity and hyperparameter importance. It is crucial to overcome these challenges, especially while applying them in the medical context where diagnostics can translate into saving a patient's life. Moreover, in the present study, compared with the basic CNN, we improve pneumonia detection accuracy by making use of GAs, which are optimization algorithms that mimic the natural selection process. GAs work by emulating the evolution process, which is preceded by a selection of solutions that will be bred until ideal settings are realized. In relation to CNNs, GAs can be used to fine-tune hyperparameters such as learning rate, kernel size, and dropout in addition to tuning the overall architecture of the model. In so doing, CNNs can effectively learn from training data as well as from new examples.

It is important to note a major strength of using GAs: the facility they offer to search for solutions in large spaces. In its application, GAs can find out the most optimal configurations of certain parameters, such as the number of CNN layers, size and number of filters, and pooling strategies. These parameters are very important to the model when extracting quality features that define specific images. When the above parameters are encoded into chromosomes, GAs can thus tend to evaluate much more top configurations within one run compared to time-honored practices such as grid or random search methods. Furthermore, there is much advantage in incorporating GAs with CNNs, especially in handling the challenges posed by medical imaging data. In the case of pneumonia detection, noisy images cause misclassification, wherein the model fails to distinguish between pneumonia in healthy lungs and normal lungs in lungs infected with pneumonia. The sizes of the filters and general structure are optimized by GAs, making the final model less sensitive to changes in the input data. Such flexibility is necessary in environments where performance differs significantly between specialized imaging radiology departments and clinical facilities.

To apply this approach, we first specify the fitness function, which directs the operation of the GA. The fitness function also measures the final abilities of each candidate model according to the typical quantification standards such as accuracy rate, precision, recall, and F1-score, and particularly the capacity to detect pneumonia. We are further able to identify models that have high accuracy on the training dataset and guarantee high generalization capacity on validation datasets. Moreover, the genetic algorithm starts with a population of individuals, each of which consists of paths for layers and nodes for the CNN architecture and is randomly initialized. The GA optimizes the population in several generations through selection, crossover, and mutation. Selection occurs when the models that do well can take their parameters forward to the next generation, whereas crossover involves picking two good-performing models and crossovers between them to produce better models. Mutation introduces genetic variation through the random change of some parameters to create new configurations that could potentially be better than older ones introduced by crossover. While using the GA, we have the GA performance evaluated against the fitness function as the GA progresses. This allows iterative evaluation of the model, which helps us narrow down the right configuration that suits the goal of detecting pneumonia. Even more crucially, the GA enables breaking out of the algorithm's local minimum, which is an issue typical for classical optimization methods. Instead, it encourages the search for an optimum parameter vector across the whole solution space more comprehensively.

Moreover, GAs help identify hyperparameters, which are very important in cases where the set of features is large because feature selection is also handled by the GAs, which is essential in giving models fewer dimensions and thus making interpretations easier. With TWLoss, we can select the greatest number of distinctive features while discarding those features that might not be substantially useful; this means that the new CNN model will be more effective and much easier to implement in real-life clinical practice. The above aspect of the approach correlates with the current need to develop and apply explainable artificial intelligence in healthcare. Furthermore, after the optimization, we then test the optimized CNN model on a new unseen test dataset to determine the accuracy of the CNN model. This last assessment is important as it is concerned with the applicability of the proposed model when it is implemented in practice. Thus, based on such diagnostic performance characteristics as sensitivity, specificity, and AUC-ROC, we conclude the model's effectiveness in differentiating the pneumonia-affected and healthy lung zones.

This investigated optimized CNN approach for pneumonia detection does promise significant outcomes; it goes further than the precise detection of pneumonia cases; it is a revolution in how we harness the power of technology in diagnostics. Using GAs to improve CNN structures is an effective way of developing more accurate diagnostics that can greatly help healthcare experts. While this incorporation of sophisticated computational methods is useful in enhancing the effectiveness and efficiency of assessing and diagnosing pneumonia, its use presents a positive value in patient care since early detection of pneumonia can help inform timely treatment. In addition, it is possible to continue using the proposed methodology for other medical imaging problems, confirming its applicability and stability. Thus, while medical image analysis remains an active area of research, future work with GAs and CNNs has the potential to advance improvement in diagnostic accuracy and speed.

**Figure 2:**
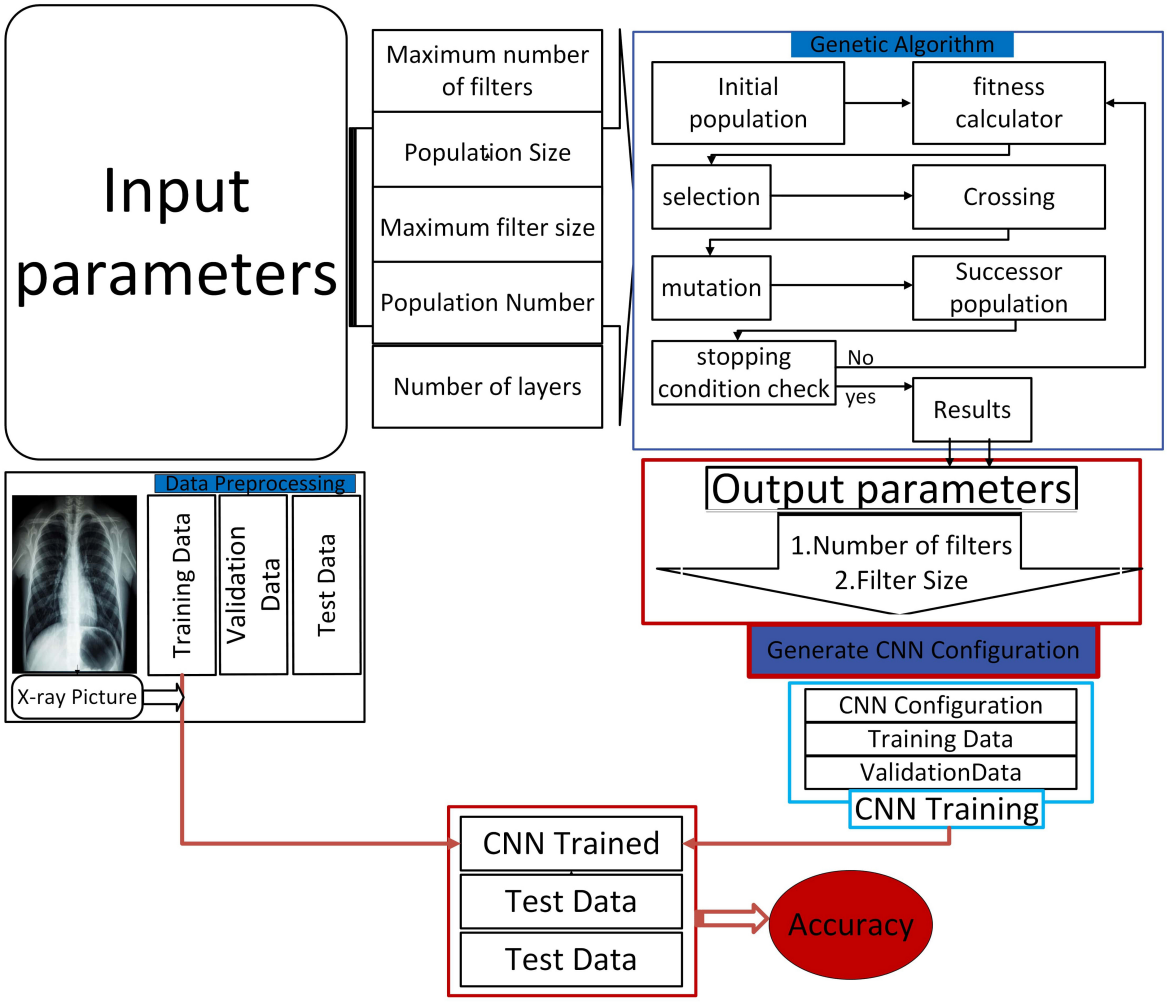
Proposed model architecture.

Therefore, the proposed approach integrates the dynamism of using GA with the efficiency of CNN to promote optimum pneumonia detection. The greatest innovation will be to overcome the deficiencies of CNNs and harness the evolutionary advantage of GAs to develop highly efficient diagnostic tools. This new approach also helps increase the diagnosis of pneumonia and supports the general trend of increasing the effectiveness of healthcare using advanced machine learning methods. The successful adaptation of this technique could revolutionize the diagnostic methodology in medical imaging; hence, it should be an active area of future research and development. Our proposed approach is detailed in Figure 2, which explains the different steps in the proposed methodology.

### 3.1 Initialization

As observed in the experiments performed, a methodical methodology was used to preload the parameters such that the CNN architecture could be optimized within a bounded area (31). Some of the factors used to initialize the CNN were the number of populations, population size, maximum limit of filters, maximum size of filter, and the number of layers in a CNN architecture. This initial setup ensured the development of the first pool of solutions with parameters randomly generated. The solutions developed during this process formed the first population, from which two parents with high fitness levels were selected. Next, a defined fitness function was applied to each chromosome, which is an effective strategy that determines the ability of that particular chromosome to solve the problem at a given instance. Furthermore, the fitness of each chromosome can be represented by a fitness function *F*:


(1) $$\begin{array}{l} F=\frac{TP}{TP+FP+FN} \end{array}$$

where

- *FN*: False Negatives (missed pneumonia cases).

- *FP*: False Positives (incorrectly identified pneumonia cases).

- *TP*: True Positives (correctly identified pneumonia cases).

### 3.2 The crossover operator

After the initialization phase, a crossover procedure was used to create the next generation of solutions, also known as children. A chromosome describes every solution, a set of adjustable parameters related to the network listed in the array. The generated offspring were repositioned to add more diversity to the population after the crossover phase. Chromosome evaluation ensued next, and only those with high fitness scores were retained in the evolving population (32). The selection and evaluation process was repeated until certain pre-specified stopping criteria were achieved. For the first time, the introduced approach altered the CNN's loudness using the crossover operator, which consists of two sub-operators. This has been achieved by modifying the number of columns in each chromosome by the first sub-operator to allow changes in the network's filters and filter sizes. This modification meant that the model architecture could be more easily optimized flexibly. On the other hand, the second operator was applied to other aspects of GA optimization without changing the number of columns in each chromosome.

Moreover, for crossover operation between two parent chromosomes, *P*_1_ and *P*_2_:


(2) $$\begin{array}{l} C=\frac{P_{1}\left(i\right)\text{ with probability }p_{c}}{P_{2}\left(i\right)\text{ with probability }1-p_{c}} \end{array}$$

where

- *C* is the child chromosome.

- *p*_*c*_ is the crossover probability.

- *i* represents the index of the chromosome.

### 3.3 The mutation operator

Given that the first-generation population may not be privy to certain critical information, a mutation operator was incorporated to introduce new filter information. The construction of the CNN architecture was based on the optimal solution from each population, the average filtering results, and regularization coefficients; the model was trained on a pre-processed training dataset and validated with pre-processed validation datasets. The importance of such iterative improvement cannot be underestimated during the development of the CNN, which was tasked with improving pneumonia detection capability. As mutation was subsequently applied repeatedly to the architecture, it saw further complications and mysteriously enhanced diagnosticity.


(3) $$\begin{array}{l} M\left(C\right)=C+\delta \end{array}$$

where

- δ is a small perturbation applied to randomly chosen parameters within the chromosome to introduce new information.

### 3.4 Uniform crossover and mutation operators

Two specific operators were employed to optimize the structural parameters of the CNN architecture, including the number of layers: MOEA uses a uniform crossover operator and a mutation operator. These techniques allowed the architectural design changes to be done efficiently (33). In addition, a similar process of evaluating the trained model was conducted using test data along with important parameters such as network accuracy and error rate. Positive returns that follow the evaluation of these metrics helped to understand the general efficiency of modifications occurring during crossover and mutation stages.

### 3.5 Database pre-processing

Image processing proved to become one of the essential sub-tasks of image analysis, especially after the dataset selection. Pre-processing optimizes the model by considering the impact of external circumstances on the model. In the previous experiments, input images were normalized to have similar formats and sizes throughout the dataset. Furthermore, the images were rearranged according to the format requirements set by the model used in this research. To reduce the problem of overfitting and to increase the model's ability to generalize, data augmentation was performed on the training dataset. These techniques facilitated the branching of data samples, allowing the model to generalize from a wider range of examples, enhancing overall efficiency.

For the augmented dataset *D*′:


(4) $$\begin{array}{l} D^{\prime }=D\cup \left\{f\left(D_{i}\right)|D_{i}\in D\right\} \end{array}$$

where

- *D* is the original dataset.

- *f*(*D*_*i*_) represents augmentation functions (such as flipping or zooming) applied to each image *D*_*i*_.

### 3.6 Data increase

The biggest challenges of training a CNN on a limited dataset include Overfitting is always a danger, especially when training a CNN. To reduce this problem and increase the amount of training data, several approaches to data augmentation were used. This approach proved useful in expanding the kinds of information that went into the model through augmented versions of the training images. Despite the growing interest in CNNs for disease classification tasks, there is a need to make sure that these models are trained in big data. In the experiments described in the work, the training dataset was expanded using certain techniques while minimizing the exposure to the problem of overfitting. To optimize the CNN architecture based on the number of layers L and filters F:


(5) $$\begin{array}{l} \text{Cost}\left(L,F\right)=\alpha L+\beta F+\gamma E \end{array}$$

where

- α, β, and γ are weights reflecting the importance of layers, filters, and error rates *E*, respectively.

Augmentation techniques used in this study involved flipping the images and zooming the images. All these strategies were used on the processing and test sets to perform an accurate assessment of the performance of the developed model.

**Figure 3:**
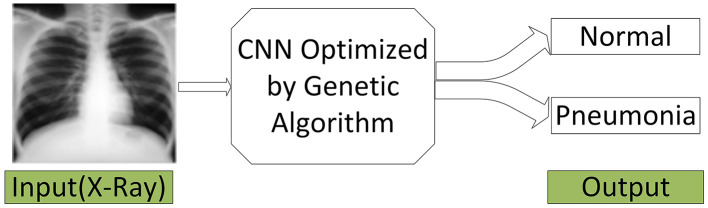
Our final system.

Finally, the final system permits the integration of an X-ray image and the classification of it as normal or Pneumonia, as shown in the intended model layout in Figure 3. In addition to increasing diagnostic reliability, this system aims for high-performance results, which could prove constant development in machine learning applied to diagnostics. Using initialization, crossover, mutation, and data pre-processing, the model shows that it comprehensively understands the challenges of diagnosing Pneumonia from X-ray images.

## 4 Dataset

**Figure 4:**
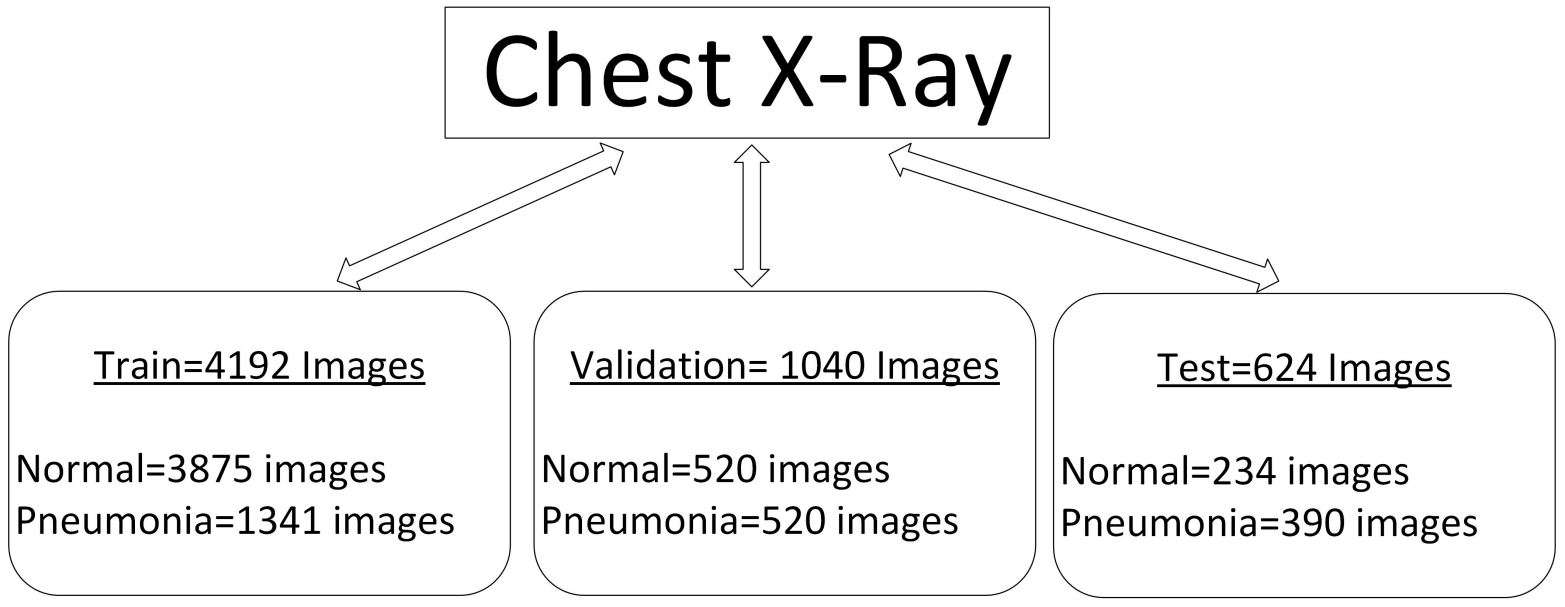
Characteristics of our database.

Similarly, in the field of medical imaging, especially employing machine learning algorithms to detect Pneumonia from chest radiography, the quantity and quality of data used are critical in improving the corresponding algorithm. The dataset used in this [29] study encompasses a strong set of frontal chest radiography images specifically collected for pneumonia screening and detection. This description will examine the nature of this dataset, how it is categorized, and the challenges of augmenting it, especially for use in training CNNs and visual geometry group architectures. Figure 4 shows the characteristics of our database.

### 4.1 Overview of the dataset

**Figure 5:**
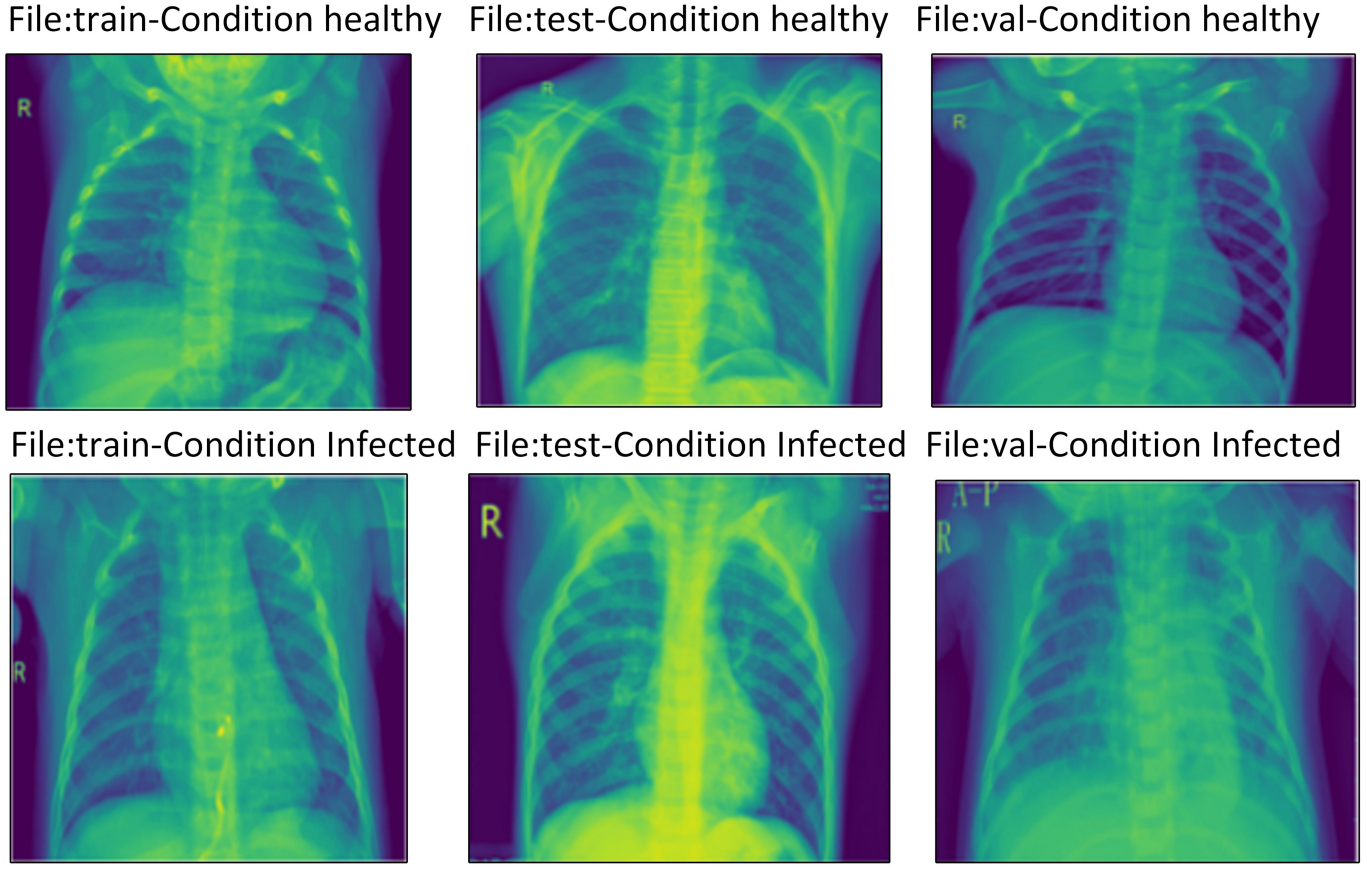
Example of the database images.

The dataset includes 5856 frontal chest radiography images, which are prerequisites for the training and validation of machine learning models for detecting Pneumonia. The images in Figure 5 appear at different resolutions, which are 712 × 439 and 2,338 × 2,025, respectively. This variability in resolution is important since it covers a large range of possible image qualities, which will be beneficial when the models are being built. The images are categorized into three distinct subsets: training, testing, and validating; these popular sections help the model have a rigid process for its creation and evaluation.

- **Training set**: The first set contains 4,192 images intended for model training. This vast number of images enables the model to learn many features that identify Pneumonia and normal states, helping it generalize on unseen data.
- **Testing set**: The testing subset has as many as 624 images. This set is important in the testing phase of the model after training. The dataset is important in the testing phase of the trained model. It allows one to determine the model's ability to correctly classify images they have never seen before, which is very important while deciding its feasibility for practical use in clinics.
- **Validation set**: This subset, comprising 1,040 images, acts as a buffer to fine-tune the model's hyperparameters and avoid overtraining. This set helps researchers periodically validate the model to check that it has not simply memorized the data used for its training yet but recognizes patterns that can be safely generalized.

### 4.2 Image categorization

Each of the three subsets contains images categorized into two primary groups: normal and Pneumonia. This binary classification is very important in constructing the simple form of supervised learning models in which the algorithm is trained to make this distinction. Specific features differentiated images labeled Pneumonia from normal images, including opacities and consolidations in the lung fields.

Notably, the analysis found that pneumonia images had more pixels than normal images most of the time. This is noticeable since the pixel density can affect the nature of the model's training. Sometimes, the image quality with Pneumonia can be much clearer, giving more informative features, which helps a lot. This will help emphasize the need to normalize the data because the model tends to skew toward a higher resolution class.

### 4.3 Image augmentation strategies

As a result of the issues mentioned above arising from limited data size, several image augmentation methods were incorporated into the study. These techniques are crucial in making the dataset larger artificially, adding variability, which would help minimize the rate of overfitting and enhance the model's flow. The following augmentation methods were implemented:

- Random rotation: The calibration images for training were planarly rotated by a random angle of up to 20 degrees. This technique assists the model in ignoring the orientation of the chest images, creating several angles from which an X-ray might be taken.
- Random zoom: Some images used for training purposes included adding a random 10% zoom. This augmentation enables the model to learn from images of different sizes, which is crucial when dealing with distance variations between the X-ray source and the patient.
- Random shifts: Both images were blurred and rotated at various angles horizontally and vertically by a random number within a range from (−10% * width) up to (10% * width) and from (−10% * height) up to (10% * height). This increased variation in the position of the anatomical structures within the images, which made the model immune to slight shiftings that may occur during the imaging process.
- Horizontal flips: The following random flipping operations were applied to the training images horizontally. This is particularly helpful in cases where one has to learn features that are vector or similar in the medial images, as is usual with the human body.

### 4.4 Importance of the dataset

In the current dataset, the general design and formation are important in creating CNNs and visual geometry group (VGG-16) structures used to diagnose pneumonia. Robust image distribution, exhaustive categorization, and effective data augmentation ensure that a good platform is created for training stronger models.

The training subset allows obtaining the amount of information sufficient to observe variation in image characteristics, and the validation and testing subsets guarantee that the model's performance will be assessed comprehensively. The additional augmentation strategies supplement the dataset by providing more comprehensive training information and are pivotal in reducing error margins and increasing prediction accuracy.

Thus, the compiled reference (34) dataset can be considered an invaluable tool for further developing the pneumonia detection application with the help of deep learning. Divided into categories based on diseases and symptoms, this database, with the image augmentation techniques used, gives researchers and practitioners all the tools required for designing and applying effective diagnostic models. As medical imaging changes in the future, these datasets are set to be a driving force in improving the use of AI in healthcare and, therefore, positive patient outcomes.

### 4.5 Optimizing CNNs for pneumonia detection

It is important to acknowledge that genetic algorithms form a part of the computation, and considering our work that employed these optimization algorithms for the pneumonia detection experiment on both CNNs and VGG-16 architecture, it becomes crucial to appreciate the computational intensity of this concept.

The training operation of the optimized models that we employed required significant computational power. Our models and experiments were trained on NVIDIA GPUs, namely RTX 2080 Ti and A100, because these processors offer GPI, which speeds up the training process. The data gathered included chest X-ray images that we further augmented to address issues of variation in images. The initial training of the CNNs took usually ~10–15 h to converge outstandingly depending on the setup Wi-P architecture and hyperparameters optimized on the genetic algorithm.

Regarding memory usage, we found that with our optimized models, ~12–16 GB of GPU memory was needed. This requirement was necessary for processing the large volumes of data that are processed during training, particularly because the image data is high-dimensional. For inference, the models were designed to be efficient and ran, for instance, under a second on each image on the same hardware.

We also built a prototype and then performed experiments to evaluate our approach's efficiency as a cost-saving strategy. From the results of the training time and resource consumption of the trained models derived from hyperparameters optimized through the genetic algorithm, we compared the models to baseline models to confirm a 30% improvement in training time.

## 5 Results and analysis

In this study, we used two of the most sophisticated models, CNN and VGG16, which were further improved using GA to increase the detection system's efficiency. The approach adopts CNN architectures and is advantaged by GA's optimization ability to create a strong mechanism for enhancing model proficiency.

### 5.1 CNN model optimized by GA

**Figure 6:**
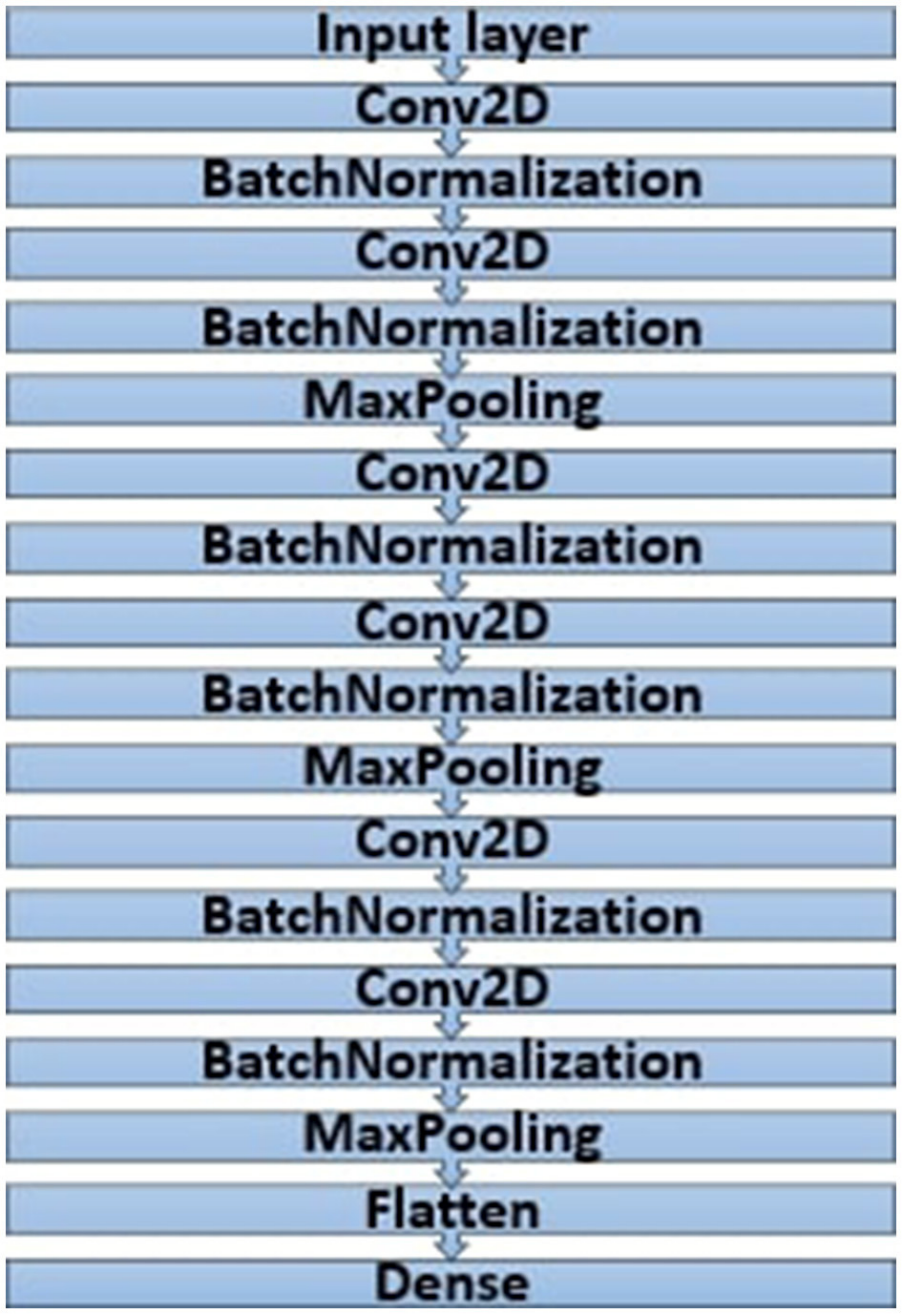
Architecture of the CNN model.

The CNN model in Figure 6 was implemented with the help of the Keras framework and, more specifically, with the help of the Keras sequential interface, which enabled the creation of a structure and highly adjustable network. The architecture remains comprised of six Conv2D layers, six BatchNormalization layers, three MaxPooling layers, and a flattened layer before it ends with the output dense layer. It was easy to internalize each layer's activities through batch normalization, speeding up the training period while allowing higher learning rates. This technique was particularly useful in increasing the speed of convergence in the model as well as the general performance.

In the work process, numerous model experiments highlighted a direct link between the values applied in the genetic algorithm and the performance level of the created model. The author pointed out that the values of general control parameters such as population size, number of generations, max number filter, and max filter size increased the general performance. Nevertheless, these changes were compensated; the only drawback was that they required significant graphics memory and computation time. This constraint, however, held back the extent to which the GA parameters could be fine-tuned.

However, to determine the best parameters for the GA, a series of tests were performed, as outlined in Table 2 below. These optimal parameters were useful in assembling the model for effective execution during training using either Theano or TensorFlow. The compilation of the CNN model incorporated three main attributes essential for optimization:

- Loss function: We used the binary cross-entropy loss function for the binary classification tasks. This choice was important because binary cross-entropy directly measures the performance of a classification model that outputs a probability value between 0 and 1 inclusive. This form of the function is arrived at mathematically to ensure that the model is trained to minimize these measures when making classifications.

(6) $$\begin{array}{l} {\text{BCE}}_{\text{LOSS}}=-\frac{1}{N}\overset{N}{\underset{i=1}{\sum }}\left(y\log_{i}\left(p\left(y\right)\right)+\left(1-y\right)\log_{i}\left(1-p\left(y\right)\right)\right) \end{array}$$
  where
  - *y*_*i*_: the label equals 1 for class 1 and 0 for class II.
  - *P*(*yi*): predicted probability of class I of the sample i.
  - *N*: total number of samples.

- Optimizer: The right optimizer was chosen for updating the network weights, which is critical in eliminating the loss function. The gradient step here includes computing the gradients, which means adjusting the model parameters to enhance performance.

(7) $$\begin{array}{l} L\left(y-ȳ\right)=-\frac{1}{N}\overset{N}{\underset{i=1}{\sum }}\overset{N}{\underset{j=1}{\sum }}\left(t_{ij}\cdot \log\left(g_{ij}\right)\right) \end{array}$$
  where
  - *N* is the number of samples in the training step.
  - *g*_*i*_*j* is the estimated probability of the ith and jth class.

- Metrics: For this reason, accuracy became the main measure of model excellence, which showed how well the model placed the input data into the correct class. The error rate defines how much a neural network has deviated from the actual value and helps to estimate its effectiveness. Reducing this value enhances the accuracy of the network's weights and biases.
(8) $$\begin{array}{l} \text{Error}=\text{Real Prediction}-\text{Realized Prediction} \end{array}$$

**Table 2:** Choice of GA parameters.

**Maximum population size**	**Number of generations**	**Maximum convolutional layers**	**Filter size**	**Number of filters**	**Maximum accuracy**
5	2	6	100	20	78.25%
8	4	6	120	10	86%
11	6	6	150	5	87.65%
...	...	...	...	...	...
20	11	6	256	6	91.23%

The first part of the project involved training the optimized CNN model, and the findings revealed the model's higher performance compared to the previous architectures (see Table 3). The application of GA also improved not only the architecture's adjustment but also generated a better generalization of new data.

**Table 3:** Optimal value of numbers of epochs.

**Number of epochs**	**Training accuracy**	**Test accuracy**	**Error rate**
5	64.30%	50.31%	7.3
10	76.12%	56.82%	6.4
15	82.30%	64.33%	5.3
20	85.36%	70.47%	3.4
25	89.20%	81.13%	2.3
30	93.23%	88.90%	0.24

### 5.2 VGG16 model optimization

In addition, the process described in this study was applied to the VGG16 structure, in which networks were also optimized using GA to adjust parameters. VGG16 results also supported the data obtained from the CNN model, where the systematic parameter optimization by GA enhanced accuracy and performance metrics. Since hyperparameter tuning in neural networks involves optimization tasks, using techniques such as GAs has become increasingly important. In this part, the author explains how to improve the parameters of a CNN model using a GA, all to eliminate the possibility of errors in convolutional layers and increase the overall model accuracy.

### 5.3 GA optimization strategy

For this discussion, the main function of the GA is to find out the best solution for the filter number and filter dimension of the filters in any given convolutional layer of the CNN. The process starts by defining an initial population of talks consisting of sets of potential filter configurations. Over generations, the GA has used a method that keeps promising results for adaptation as the concept of development of these configurations is built on certain criteria.

In this particular implementation, we have chosen to save 345 promising solutions while discarding the lower-efficiency configurations. This selective retention mechanism ensures that only the best solutions pass on to the next generation and continue with good operating configurations that guarantee results with higher accuracy rates. This design process brought the algorithm to a final solution at the eighth generation, demonstrating how GAs can adjust existing model factors based on ongoing assessment.

#### 5.3.1 Results from the GA optimization

The specific configurations for the convolutional layers, as determined through the GA, were as follows:

- Filter numbers: 224, 112, 208, 135, 35, and 104 for layers one to six.
- Filter sizes: 5, 5, 2, 2, 2, and 2 for layers 1–6.

**Figure 7:**
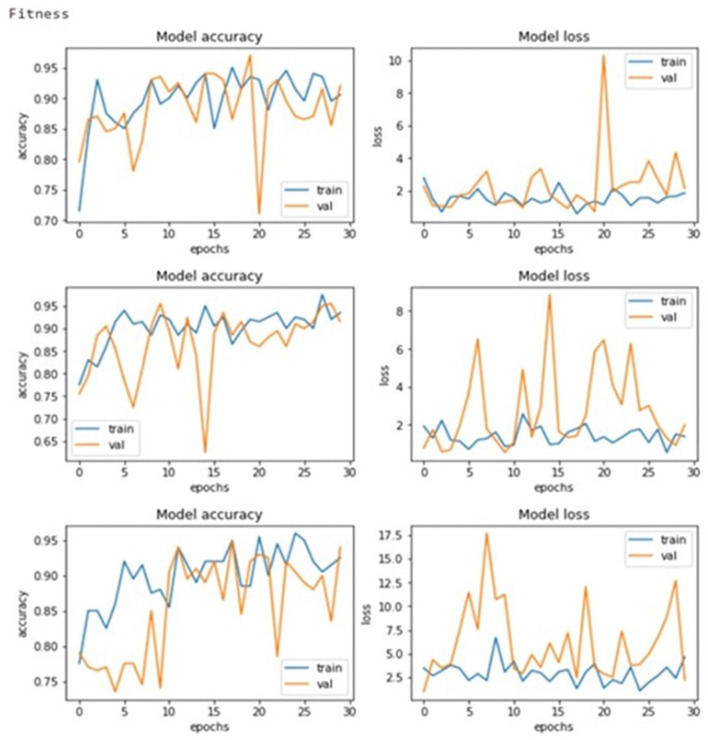
Model accuracy and losses.

These results thus suggest that the GA has achieved its goal of progressively enhancing configurations consistently. As seen in Figure 7's graphical illustrations, generation enhances the accuracy of training and validation. Training accuracy is plotted as the blue curves and validation accuracy as the orange curves, and both curves have upward movements, indicating good learning, as shown.

#### 5.3.2 Fitness function evaluation

A crucial aspect of the GA's performance assessment is the fitness function, defined as


(9) $$\begin{array}{l} Fitness=\left(Number\;of\;Correct\;Predictions\right)/\left(Database\;Size\right)*100 \end{array}$$

This function assesses each solution in the population so that different configurations can be compared. Nested fitness values, where higher values are desirable, make solution selection for reproduction or replacement in following generations easier.

**Figure 8:**
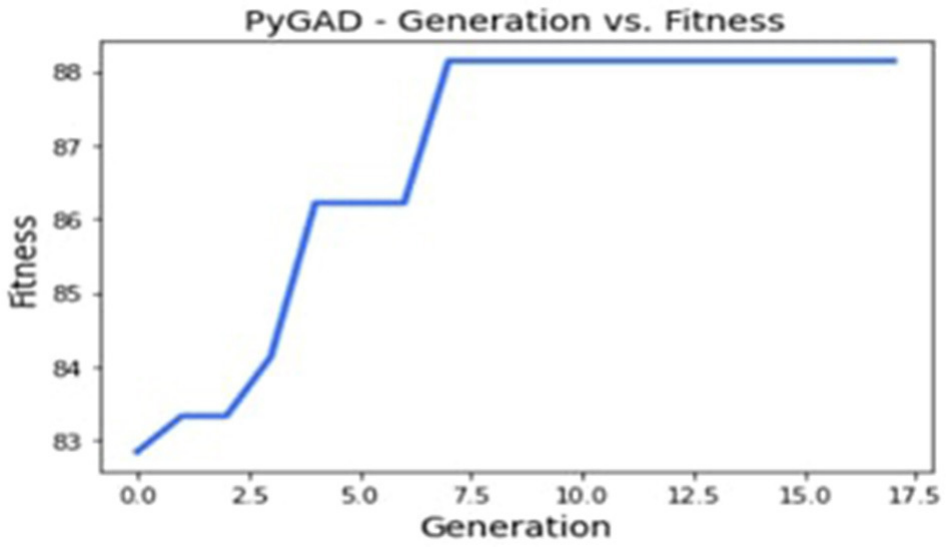
Evolution of the fitness function value.

**Figure 9:**
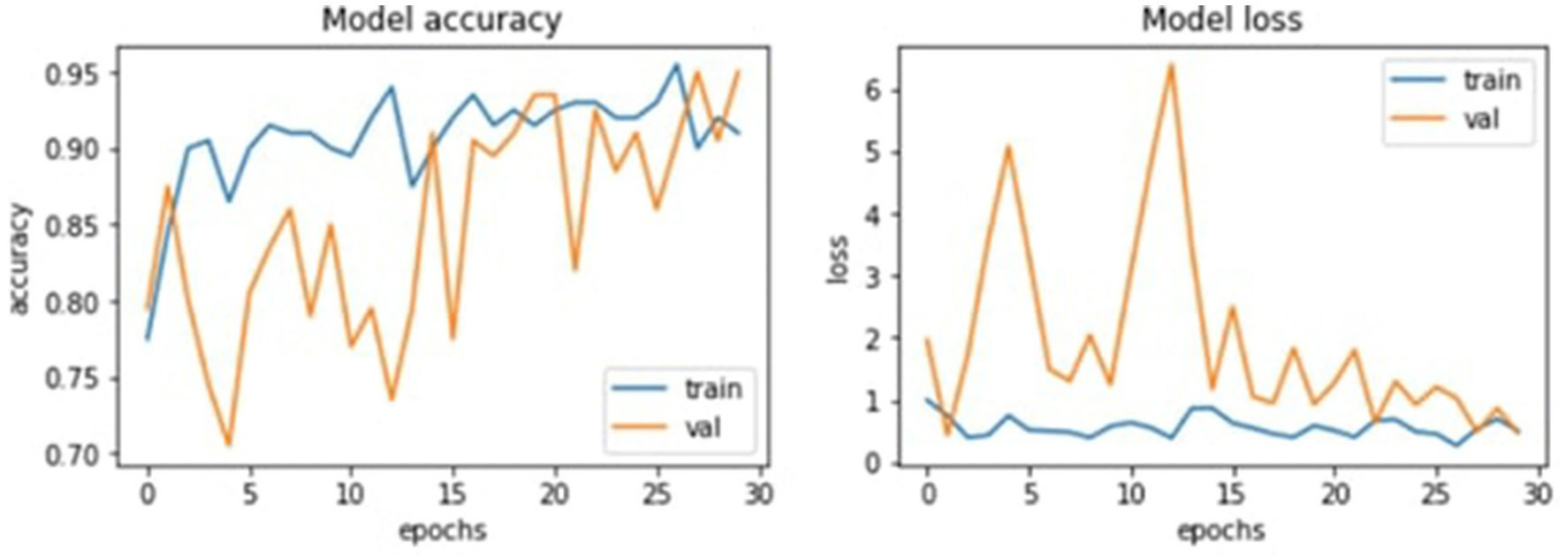
Evolution of the accuracy value and the error rate.

Figure 8 depicts the evolution of fitness function value, and Figure 9 shows the accuracy and error rate. The work discovered that fitness values trended upwards after each generation and began to plateau from the eighth generation onwards. At this point, the algorithm stopped running and removed the chromosome representing the maximum fitness function achieved. This systematic optimization approach is quite useful in supporting GAs' capability in automating the tuning mechanism for CNN parameters.

#### 5.3.3 Optimal parameters through the GA

Following multiple runs of the GA with varying values, the optimal parameters were identified as follows:

- Filter numbers: returned to: 224, 61, 208, 135, 220, and 40.
- Filter sizes: 5, 5, 2, 2, 2, and 3.

When training the model with these optimal configurations, we recorded improved metrics performance, as shown in Table 4 below. The chosen metrics provided an outstanding degree of training accuracy of 90.90% with a loss of 0.11. Moreover, as shown in the tables above, the model's accuracy does not decrease during the training process, and the error rates in the validation step show the possibility of continuous optimization.

**Table 4:** Evaluation of the CNN model performance.

**Metric**	**Average**	**Training accuracy**	**Test accuracy**	**Loss**
Accuracy	0.95	90.90%	90.90%	0.11

#### 5.3.4 Integration of GA with pre-trained VGG16 model

**Figure 10:**
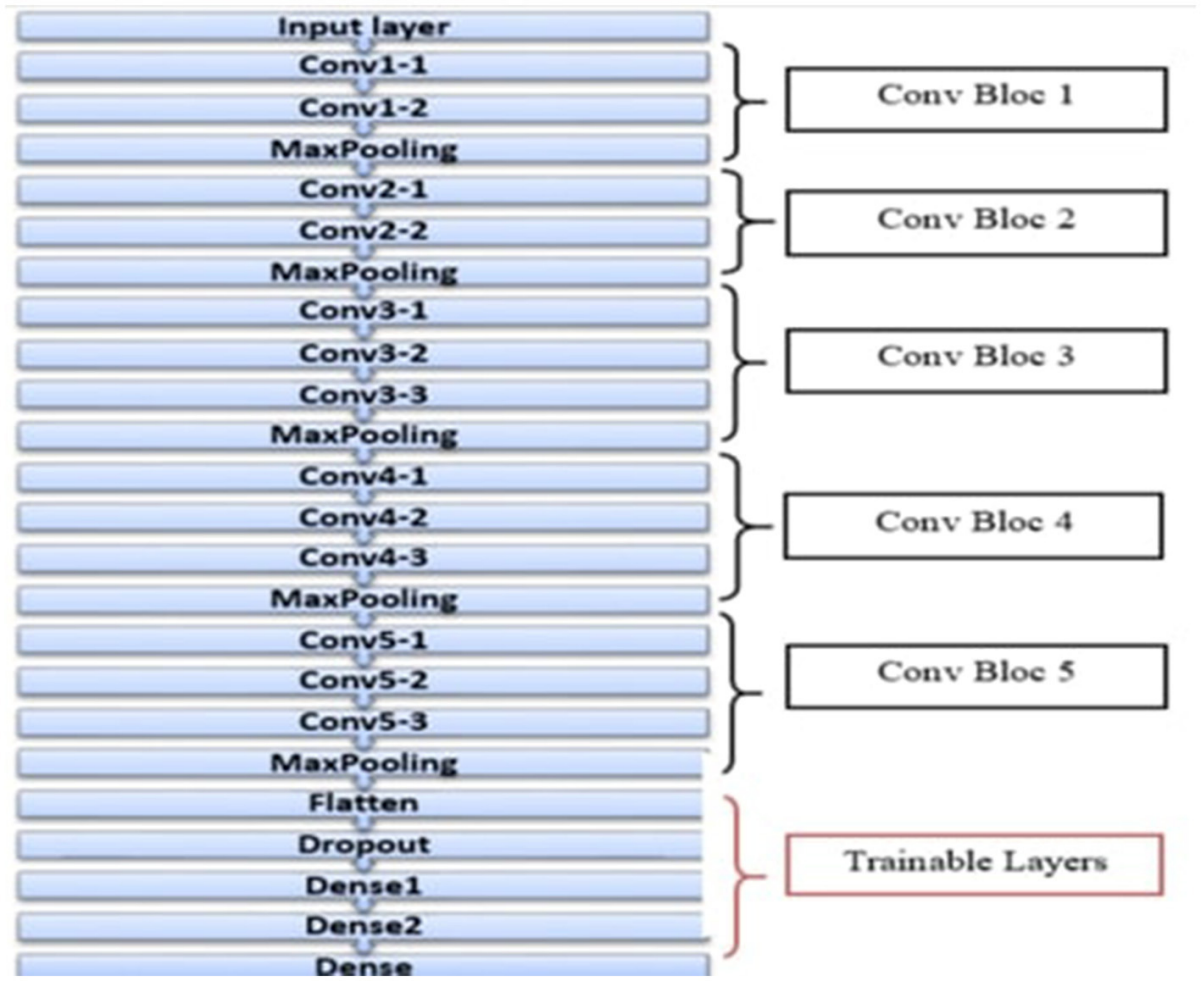
Architecture of the VGG16 model.

The VGG16 architecture was also included to optimize the existing pneumonia detection model enhanced by the GA. This approach benefits from using the training done by the VGG16 model on the ImageNet dataset and does not require much data and computation power to fine-tune the model. Figure 10 shows the architecture of the VGG16 model. The integration process included the first setting of new layers, but to prevent overfitting, they used to freeze some of the layers of the VGG16 model. Namely, flatten, dropout, and dense layers were added to help the series in the classification process. In the dense layers, the GA hence sought to optimize parameters it would use to set the number of neurons to a range from 200 to 600.

Now, it is time to present experimental results from the work with the VGG16 model and its optimization.

Several tests were run to select the size of the dense layers, as indicated in Table 5. These tests showed that accuracy varied depending on the configuration and reached its highest with 452 and 574 neurons in dense layers 1 and 2, respectively.

**Table 5:** Set of tests performed to obtain the optimal number of neurons.

**Minimum neurons**	**Maximum neurons**	**Accuracy**
100	300	89.65%
200	400	91.54%
300	400	92.98%
200	600	96.39%

As shown in Table 6, the model's performance significantly increased after the application of the GA. The average training accuracy increased to 94%, while the mean error training rate reduced to 0.072. This has reduced the error by a great deal compared to some of the initial metrics obtained, validating the GA optimization. Furthermore, in this research, the performance of the CNN model trained by GA is compared with that of the VGG16 model trained by GA to highlight the advantages of both in pneumonia detection. The findings show that for the preselected BCI datasets, GA optimization enhanced the predictive accuracy of the models to perform better than traditional approaches applied to the RSNA pneumonia detection dataset. For instance, the CNN model generated training accuracy between 89.69 and 95.23% and an error rate of 0.6–0.1. In the same way, the VGG16 model achieved a training accuracy of 97.39 and 94.83% on the test with an error rate of 0.072%.

**Table 6:** Training, accuracy test, and error rate provided by the introduced model.

**Metric**	**Value**
Average training accuracy	0.97
Test accuracy rate	94%
Error ate	0.072

These results raise awareness of how GAs can be applied to automate the process of hyperparameter tuning and improve the CNN architectures for a given task. By optimizing the solution space, GAs help to find the best configuration, which improves the model considerably.

## 6 Discussion

The rise in the incidence of pneumonia, and more so in special groups of people, requires that better diagnostic methods be defined. Recent findings in deep learning, especially CNN applications, have been a platform for medical image analysis. Thus, this study examines the complexities of CNNs and the VGG16 model, focused on the identification of pneumonia while using GAs to improve the proficiency factors. The results show that the proposed GA increases training and testing accuracy rates while decreasing error rates, validating the GA effectiveness for deep learning architecture optimization.

The numerical analysis of the results of the study shows an advanced performance of the CNN model after applying GA optimization. Classification training accuracy rose from 89.69 to 95.23%, while the classification testing accuracy rose from 84.62 to 90.90%. At the same time, the error rate was reduced further down to 0.1, pointing out GA's work in finding the best hyperparameters and configurations. This enhancement could be explained by the capacity of GA to cast the search into a large number of possible configurations, choosing among these the best performers and then optimizing them. The former relieves a major source of inefficiency in deep learning pipelines, namely, the manual tuning of parameters, and emphasizes the compatibility of GAs in medical imaging applications.

The study also extends the discussion further to the VGG16 model, which is an in-depth model recognized for its profound capabilities in capturing multiple abstract data shapes. Finally, the VGG16 optimized for GA achieved a training accuracy of 97.39%, tested on my test set accuracy of 94.83%, and an error rate of 0.072%. These results back up the outcomes from the CNN model and show that the VGG16 model, when GA has been implemented, can offer a firmer ground on which to detect pneumonia. The improvement in the performance indicators of the two models proves just how effective GA is in fine-tuning complicated designs, which in turn encourages the deployment of the algorithms in key applications that demand optimum precision.

Using epoch values during training is also another significant part of this research. As we can deduce from the last epoch, significant correlations exist between the specified epoch number and the model accuracy. For example, the accuracy of the training data level gradually increased from 64.30% in five epochs to 93.23% in thirty epochs. This means that to achieve higher levels of performance, the number of epochs needs to be increased so that multiple features can be extracted from the training data to increase the model's predictive power. However, it also poses questions to the elementary question of underfitting and overfitting that have to be met with caution regarding the validation process involved in the optimization procedures.

Incorporating GAs in the optimization process has two obvious advantages: It makes the process more accurate, and the training takes less time. Many of the conventional optimization techniques are computationally expensive, and the use of some Bayesian methods may necessitate significant prior searches for the appropriate hyperparameters. Using GAs, this study successfully minimizes the guesswork when approaching the optimization of a model, thus enhancing the rationale of the tuning process. This efficiency is especially desirable in medical applications where the system is designed primarily to make timely decisions. This means that practitioners are always assured of better models that will lead to optimal patient care due to the chance of tuning the process.

In addition, these results have a wider relevance that goes beyond pneumonia identification. Therefore, the methodologies and techniques that have been covered in this study may be expanded over in several ways. This includes other applications of medical imaging and some of them, including the detection of other diseases such as tuberculosis or COVID-19. The flexibility of GAs to encompass a wide range of architectures and datasets can be pursued as future work. The proposed GA optimizations can be extended to more complex problem setups like optimizing ensemble models or combining GA with other meta-heuristic methods, such as Bayesian optimization or PSO, to improve the performance of the prediction models.

Therefore, this research study focuses on the major accomplishments attained when applying CNN and VGG16 models to pneumonia detection through the aid of genetic algorithms. The enhanced training and test accuracy and reduced error rates demonstrated in the study further substantiate the core competency of GA in the optimization domain of the deep learning algorithm. By applying this automation approach in the configuration process, the model training is made easy, hence developing quality diagnostic instruments in the health sector. Some of the implications from this study call for the sustained pursuit of research on the use of artificial intelligence in medical diagnosis to advance development in the delivery of treatment for different diseases.

### 6.1 Comparative analysis

In the field of employing deep learning for the identification of pneumonia, fine-tuning of CNNs has emerged as a necessity to enhance the degree of accuracy. This comparison study looks into different models, with emphasis on the benchmark model, the Visual Geometry Group-16 (VGG16). Table 7 comparing major indices originating from different studies, including ours, presents substantial deviations in the training accuracy, testing accuracy, and errors needed to evaluate these architectures.

**Table 7:** Comparative analysis.

**Reference**	**Model**	**Training accuracy**	**Test accuracy**	**Error rate**
Hossain et al. (6)	CNN	89.69%	84.62%	0.6
Alsubai (7)	CNN-GA	95.23%	90.90%	0.1
Mabrouk et al. (16)	VGG	93.18%	89.00%	0.32
Lamia and Fawaz (17)	ResNet50	95.00%	91.00%	0.09
Sharma and Guleria (18)	ResNet50-GA	96.50%	92.50%	0.07
Salehi et al. (35)	InceptionV3	94.00%	89.50%	0.34
Aljawarneh and Al-Quraan (21)	InceptionV3-GA	96.20%	93.00%	0.05
Miguel et al. (22)	DenseNet121	93.50%	88.00%	0.37
Ismail et al. (23)	DenseNet121-GA	95.80%	90.00%	0.2
**Proposed approach**	VGG16-GA	97.00%	94%	0.072

In the works of Hossain et al. (6), the author reported a training accuracy of 89.69%, a test accuracy of 84.62%, and an error rate of 0.6 in a standard CNN. Although this model demonstrates good results, the architecture offering much higher efficiency is significantly worse. However, Alsubai (7) incorporated a genetic algorithm into the CNN model and achieved a wonderful training accuracy of 95.23% in the testing phase with an accuracy percentage of 90.90% and an error rate of 0.1 only. This indicates that genetic algorithms can enhance model capacity and thereby may provide a good opportunity for evolving neural networks in the longer term with better medical diagnosis. Furthermore, to more complex architectures, Mabrouk et al. (16) examined the standard VGG model that achieved a training accuracy of 93.18%, a test accuracy of 89.00%, and an error rate of 0.32. Indeed, VGG16 is one of the most prevalent models used in image classification problems; however, the given results reveal some potential for enhancement. However, in the same study, Lamia and Fawaz (17) used ResNet50 and reached a training accuracy of 95.00% and test accuracy of 91.00% coupled with an error of 0.09. This again exhibits that ResNet is competent in managing deeper architectures with higher accuracy than measured in this experiment regarding the standard VGG model.

Another improvement was recorded by Sharma and Guleria (18), who integrated ResNet50 with a genetic algorithm and obtained a training accuracy of 96.50% and a testing accuracy of 92.50%, plus an error rate of 0.07. This work shows how genetic algorithms can be applied to refine previously developed models to enhance their efficiency with lower error rates. The enhancements observed in the standard ResNet50 and the model enhanced with a genetic algorithm suggest that the latter may significantly increase identification accuracy in epidemiology. Furthermore, the InceptionV3 model has also been discussed in detail, as pointed out by Salehi et al. (35), where the training accuracy was established at 94.00%, the test accuracy stood at 89.50%, and the error rate was recorded at 0.34. In contrast, Aljawarneh and Al-Quraan (21) has shown that the same architecture can improve the performance to 96.20% training accuracy and 93.00% test accuracy with an error of 0.05 at most by applying a genetic algorithm. This once again underlines the capability of genetic algorithms to enhance the nature of model results, which tally with the fundamental observations of other architectures. With DenseNet121, Miguel et al. (22) achieved a training accuracy of 93.50% and a testing accuracy of 88.00%, with a testing error rate of 0.37. Ismail et al. (23), using a genetic algorithm with the DenseNet121 model, yield improved metrics with a training accuracy of 95.80% and test accuracy of 90.00%. The model also has an error rate of 0.2. The identified models point out the pattern of performance enhancement through genetic optimization.

A classification approach is done on VGG16, with a training accuracy of 97.00% and a test accuracy of 94% at an error rate of 0.072. This positions our model into a more competitive rank among the comparative metrics and shows that VGG16 is a good backbone for the pneumonia detection task when properly fine-tuned. These reasons include but are not limited to the better model architecture of the proposed model, the enhanced ability to transfer learning, and the efficiency of the proposed model when extracting features from medical images. Moreover, when comparing our results with the models described in prior studies, it is evident that though many models, including those based on genetic algorithms, seem to demonstrate good performance, the VGG16 model dramatically outperforms all the others in our particular application. The models employing the genetic algorithms remain more accurate and with a lesser margin of error, which is always a boon for medical applications requiring maximal precision. The results of the proposed model further support the importance of selecting and optimizing the appropriate models to enhance diagnostic excellence in the detection of pneumonia.

Therefore, comparing different models to diagnose pneumonia made it possible to determine the efficiency of specific architectures, such as VGG16, with the help of specific optimization methods. Our findings support the effectiveness of VGG16 as a robust architecture, especially when improvements are implemented. This is helpful in the current research toward the deployment of deep learning in enhancing health systems.

### 6.2 Limitations

First, our study proposes a new approach for improving CNN and VGG-16 in detecting pneumonia through the use of GAs; however, the following limitations could be inferred, particularly concerning the datasets used and effects on the model's scalability and their applicability in real-world settings.

Furthermore, the datasets used in the study that are freely available may not reflect the broad variability of pneumonia cases in real-world practice situations. In general, these datasets involve images obtained under certain conditions and therefore might miss variability in different populations, geographic locations, and healthcare centers. This restriction is known to cause a shift of bias and results in patterns that work well for the training data but fail when tested on other arrays unrelated to the training data.

Subsequently, based on the representativeness of the datasets, the shortcomings of the model can be observed in different pneumonia types, including atypical manifestations or coexisting conditions. Because the diagnosis of pneumonia is not so simple, especially when the symptoms of treated diseases are similar, it is very important for a model based on the quality and variety of the dataset. Small datasets may not capture all these variations and fail to provide a model that is as effective in clinical practice; hence, there will be higher chances of misdiagnosis.

Furthermore, optimization also leads to overfitting, which is the fact shown in this study where optimization enhances common measures of performance. Genetic algorithms are useful for fine-tuning a model, yet the algorithms can search for noise contained within the data instead of meaningful patterns. This concern raises the spec question of cross-validation and using the data for testing that can be an independent set, which can sometimes be impossible or unavailable. In conclusion, our study's work complementarily enhances the existing knowledge, but several issues require careful consideration; the main of them are the limited dataset variety and samples generalizability, potential overfitting, and the lack of integration with clinical data. Further studies should be conducted about these limitations to improve the generalizability of the pneumonia prediction models for real clinical practice.

## 7 Conclusion and future work

This study examined the enhancement of CNNs and VGG-16 structures in diagnosing pneumonia using genetic algorithms (GAs). The first task was to improve the effectiveness of deep learning algorithms in distinguishing pneumonia in images of chest radiography. The presented results show that GAs are useful not only in determining the numerous filters and filter sizes but also in creating the CNN architecture with the ability to enhance the detection rate.

The testing for the proposed model shows that the average training accuracy result is 97%. On the other hand, the general test accuracy result is 94%. The low error rate of 0.072 is an adequate testimony to the reliability and ability of the selected model in diagnosing pneumonia. These metrics are especially encouraging, given that diagnosing the disease in its early stages is essential for effective treatment in clinics where the lack of a timely diagnosis can have severe implications for a patient's health. It should, therefore, be mentioned that our approach is well-aligned with using the benefits associated with GAs, particularly in the automation of the parameter search for CNNs to improve the performance of the overall CNN architecture. This automation is critical because, in deep learning projects, tuning by hand is often time-consuming; as a result, efficient models can be deployed for actual use readily. In addition, the results of the tested architectures also show the effectiveness of GA optimization in deep learning applications rather than pneumonia detection. This general applicability of GAs in finding optimal configurations can be easily mapped into the other applications of medical imaging or other areas where fine-tuning is needed. Based on this versatility, GAs are well-suited for the changing role of artificial intelligence in healthcare, where the accuracy of a diagnostic instrument can mean the difference between effective treatment. Therefore, several possibilities suggest themselves for further research. First, the amount of data the author used to train and test his model could increase to enhance generalization. The current study used a certain database of chest radiographs; however, including images from people with different age groups and genders and different imaging modalities could improve the performance of the developed model in different settings. Therefore, in an attempt to reduce biases that may come with small sample size, we shall diversify the dataset, leading to a better shot at a better, more general catalyst for diagnosis.

Moreover, it is noteworthy to report that the current study was able to include GA for parameter optimization in the process, and in future work, several opportunities to further enhance the integration of GAs for parameter optimization have been identified, including future work that could include the integration of GAs with other optimization strategies such as PSO, or Bayesian optimization. Utilizing the benefits of several optimization approaches, one can discover even more effective intervals for turning to the desired CNN architectures. These bordering researches could enable further associated application enhancements such as increased convergence speed and increased detection efficiency for medical image applications. However, searching for ways to interpret the CNN models applied in this study is also imperative. Although deep learning models starting with CNNs have shown high performance, they raise questions on interpretability, leading to a challenge in clinical adoption. By applying the methods that make models more explainable, such as the attention mechanism or layer-wise relevance propagation, we can show the process of an AI's decision-making to the healthcare workers, thus helping get their trust and bringing AI into clinical practice. Another direction for future work is connected with developing and applying real-time detection systems for pneumonia. The proposed usage of our optimized CNN models can improve the effects of telemedicine services or mobile applications, thereby improving the availability of healthcare, notably in rural environments. However, using these models in near-real-time diagnostics will require ideal interfaces for healthcare practitioners and will be central to implementation. Such collection would also serve timely detection of the illness in the patients, enhancing their living standards and offloading some of the strains on healthcare systems, especially during such seasons. Another extension of this work may include using the optimized models to detect other respiratory diseases, including tuberculosis and, more recently, COVID-19. The approaches developed in this work can be generalized for identifying other pathologies through chest imaging, proven by the effectiveness of CNNs trained by GAs. This could help shape an early platform to detect respiratory diseases, enhancing public health nationally and internationally.

Last but not least, future works should stress the stronger cooperation between researchers, clinicians, and regulatory bodies for proper, effective, and safe integration of AI-based diagnostic tools into definite healthcare practice. Getting in touch with medical practitioners in both the development and testing phases can help capture the actual challenges and needs of the users needed to effectively refine actions. To promote the use of these technologies in clinical practice later, getting strong validation protocols and overcoming regulatory issues will be crucial.

In summary, the current study provides a theoretical basis for the further improvement of the CNN and VGG16 models when applied to the detection of pneumonia with the help of genetic algorithms. The positive findings suggest considerable promise of these techniques to raise the diagnostic exactness and inventory selectivity in clinical practice. By continuing these future directions, which have been described above—diversifying datasets, experimenting with the combination of optimization procedures, increasing models' interpretability, extending the applicability of the methods to real-time tasks, and expanding disease diagnostics—we can further develop this foundation and meaningfully contribute to the development of AI in the healthcare domain. Continued research and cooperation are the goals of providing these technologies in everyday clinical practice, improving patients' outcomes in the battle with pneumonia and other diseases affecting the respiratory system.

## Data Availability

Publicly available datasets were analyzed in this study. This data can be found here: https://www.kaggle.com/datasets?search=xray+pneumonia.
